# Editorial: Novel Insight Into the Diagnosis and Treatment of Cardio(Thoracic) Diseases in Dogs and Cats

**DOI:** 10.3389/fvets.2022.914602

**Published:** 2022-05-09

**Authors:** Lina Hamabe, Meriç Kocatürk, Zeki Yilmaz, Ryou Tanaka

**Affiliations:** ^1^Department of Veterinary Surgery, Tokyo University of Agriculture and Technology, Fuchu, Japan; ^2^Department of Internal Medicine, Faculty of Veterinary Medicine, Bursa Uludag University, Bursa, Turkey

**Keywords:** echocardiography, color Doppler, cardiac biomarker, two-dimensional tissue tracking echocardiography, RV function, pulmonary arterial wave reflection

Improved diet, management, and health care of companion animals have led to increased life expectancy, as a result, cardiac diseases have become pivotal causes of death for both dogs and cats. For such reason, a major objective has become to increase the quality of life by reducing the expansion of cardiac diseases via early detection of diseases and provision of effective treatment.

Conventional echocardiography and cardiac biomarkers, such as N-terminal pro-B type natriuretic peptide (NT-ProBNP) and cardiac troponin I (cTnI), are the most routinely applied method of diagnosing cardiac disorders. While they provide a diagnosis when cardiac dysfunction is prominent, prediction or early diagnosis of cardiac dysfunction prior to the development of overt clinical symptoms is challenging. This Research Topic aims to provide the recent trends in the diagnosis and treatment of cardiothoracic diseases in dogs and cats. It presents 9 peer-reviewed research articles, which explore the diagnosis and treatment of cardiothoracic diseases with emphasis on novel echocardiographic techniques and bio-fluid analysis of cardiac biomarkers.

Two-dimensional speckle tracking echocardiography (2D-STE) is an advanced echocardiographic technique that allows comprehensive analysis of myocardial function by quantifying myocardial deformation. There is growing evidence that the parameters of this deformation analysis are superior to the conventional parameters for the early diagnosis of myocardial dysfunction (1). Suzuki et al. evaluated the relationship between congestive heart failure (CHF) and myocardial function in cats with cardiomyopathies using 2D-STE. The results revealed left atrial enlargement and decreased left ventricular apical circumferential strain in cats with CHF, suggesting a possible association with progression from sub-clinical cardiomyopathy to CHF. Furthermore, evaluation of the right ventricle (RV) demonstrated increased end-diastolic RV internal dimension and decreased RV longitudinal strain in cats with CHF, also indicating an association with the onset of CHF in cats with cardiomyopathy.

In recent years, the importance of RV function has become apparent, and it is now known to play a critical role in the pathophysiology of various cardiovascular diseases. Yuchi et al. evaluated the RV myocardial adaptation associated with increased pulmonary arterial pressure (PAP) in a canine model of chronic pulmonary hypertension (PH) using 2D-STE. In the acute phase of PH, temporal reduction of RV longitudinal strain was observed as a result of acute rise in PAP, which improved with progressive RV hypertrophy, indicative of RV adaptive remodeling. In the chronic phase, RV dilation and reduction in RV longitudinal strain were observed, suggestive of RV maladaptive remodeling and myocardial dysfunction. Such results suggest that the RV longitudinal strain is reflective of the intrinsic RV myocardial contractility during the PH progression, which could not be detected by conventional echocardiography.

Pulmonary arterial wave reflection (PAWR) analysis, which can be performed non-invasively via wave intensity analysis of the Doppler echocardiography, is a novel technique that gives valuable information on pulmonary artery hemodynamics in PH (2). Yoshida et al. investigated the changes observed before and after mitral valvuloplasty in dogs with PH caused by myxomatous mitral valve disease (MMVD) using PAWR, which demonstrated mitral valvuloplasty as an effective treatment of PH secondary to MMVD.

Cardiac biomarkers are of great importance in the early diagnosis and treatment of cardiac diseases, which include but are not limited to metabolic by-products, enzymes, proteins, peptides, and microRNAs (miRNAs). A prospective, multicenter case-control study by Valente et al. measured the plasmatic concentration of asymmetric dimethylarginine (ADMA) and symmetric dimethylarginine (SDMA) in dogs with various stages of MMVD. The results revealed that ADMA, a biomarker of endothelial dysfunction, was significantly associated with LA enlargement, whereas SDMA, a biomarker of renal dysfunction, was significantly correlated with serum creatinine level.

The intestinal microbiota is known to play a key role in the physiological process of mammals, and alteration of intestinal microbiota has been associated with various diseases (3). Araki et al. compared the intestinal microbiota between dogs with MMVD and healthy dogs using 16S rRNA gene amplicon sequencing. While echocardiographic and radiographic parameters showed significant differences, diversity, and composition of intestinal microbiota showed no significant differences among the groups.

Proteomic and peptidomic analysis allow evaluation of changes in protein and peptide compositions, and previous study on serum proteomics in dogs with MMVD has shown that dogs with different stages of CHF showed different serum protein compositions (4). Similarly, Petchdee et al. investigated serum peptidomics in dogs with MMVD, which demonstrated the presence of peptides including mitogen-activated protein kinase, kallikrein, and tenascin-C in the group with progressed MMVD. Additionally, Sukumolanan et al. compared serum peptidomic profile of cats with sarcomeric gene mutations and normal cats, and revealed that expression of three peptides, including FOXO1, CYP3A132 and AGAP2, were increased in cats with sarcomeric gene mutations.

Altered expressions of circulating miRNAs have been reported in various cardiac diseases in humans, and anticipated as novel cardiac biomarkers as they can be found in serum or plasma in a stable form (5). A study by Ro et al. investigated circulating levels of 11 miRNAs in serum samples of dogs with cardiac diseases, which found that cfa-miR-130b was able to accurately distinguish dogs with cardiac diseases from healthy dogs. Sukumolanan et al. developed a loop-mediated isothermal amplification assay (LAMP) coupled with a lateral flow dipstick (LFD) test to detect myosin-binding protein C3 A31P (*MYBPC3-A31P*) missense mutation in Maine Coon cats, which is a genetic deviation associated with the development of hypertrophic cardiomyopathy (HCM). This novel test was able to distinguish between cats with *MYBPC3-A31P* wild-type and *MYBPC3-A31P* mutant-type from blood samples, which has excellent potential as a novel screening test.

This Research Topic of studies has illustrated the current understanding of the diagnosis and treatment of cardiothoracic diseases in dogs and cats. We hope that these articles will inspire and encourage further studies on the advancement of novel echocardiographic techniques and bio-fluid analysis of cardiac biomarkers.

## Author Contributions

All authors listed have made a substantial, direct, and intellectual contribution to the work and approved it for publication.

## Conflict of Interest

The authors declare that the research was conducted in the absence of any commercial or financial relationships that could be construed as a potential conflict of interest.

## Publisher's Note

All claims expressed in this article are solely those of the authors and do not necessarily represent those of their affiliated organizations, or those of the publisher, the editors and the reviewers. Any product that may be evaluated in this article, or claim that may be made by its manufacturer, is not guaranteed or endorsed by the publisher.
